# Factors associated with the retention of secukinumab in patients with axial spondyloarthritis in real-world practice: results from a retrospective study (FORSYA)

**DOI:** 10.1136/rmdopen-2022-002802

**Published:** 2023-03-15

**Authors:** Maxime Dougados, Julien Lucas, Emilie Desfleurs, Pascal Claudepierre, Philippe Goupille, Adeline Ruyssen-Witrand, Alain Saraux, Anne Tournadre, Daniel Wendling, Cédric Lukas

**Affiliations:** 1 Rheumatology, University of Paris, Hopital Cochin, Paris, France; 2 Biostatistics, RCTs, Lyon, France; 3 Medical Affairs, Novartis Pharma SAS, Rueil Malmaison, France; 4 Rheumatology, Hopital Albert Chenevier, Creteil, France; 5 Rheumatology, Université de Tours, Tours, France; 6 Centre d'Investigation Clinique de Toulouse CIC1436, Inserm, University of Toulouse 3, Rheumatology Center, Toulouse University Hospital, Toulouse, France; 7 Rheumatology, Université de Bretagne Occidentale, Brest, France; 8 Rheumatology, Université Clermont Auvergne, Clermont-Ferrand, France; 9 Rheumatology, CHRU de Besançon, Besançon, France; 10 Rheumatology, Université de Franche-Comté, Besancon, France; 11 Rheumatology, University of Montpellier, Montpellier, France; 12 University Hospital Centre Montpellier, Montpellier, France

**Keywords:** antirheumatic agents, biological therapy, spondylitis, ankylosing, tumor necrosis factor inhibitors

## Abstract

**Background:**

Secukinumab efficacy and retention data are emerging in patients with axial spondyloarthritis (axSpA) in real-world settings. However, limited data are available on the predictive factors that affect the retention rate. The key objective was to determine whether objective signs of inflammation (OSI) were predictive of secukinumab retention at 1 year.

**Methods:**

FORSYA is a French, multicentric, non-interventional, retrospective study in adult axSpA patients who received secukinumab treatment between its launch (11 August 2016) and 31 August 2018. The time to secukinumab discontinuation and retention were analysed using a Kaplan-Meier (KM) analysis. OSI was predefined by at least one of the criteria: C reactive protein ≥5 mg/L or erythrocyte sedimentation rate ≥28 mm/hour at secukinumab initiation or MRI inflammation at the sacroiliac or spine level.

**Results:**

In total, 906 patients from 48 centres were included in the analysis, 42.2% of whom were men, with a mean age of 46.2±11.7 years and a mean disease duration of 9.3±9.1 years. The 1-year KM retention rate (95% CI) for secukinumab was 59% (55%–62%), whereas for patients with and without OSI, it was 58% (54%–62%) and 63% (53%–73%), respectively. In multivariate analysis, lack of prior exposure to tumour necrosis factor inhibitor (TNFi), absence of OSI and inflammatory bowel disease (IBD) were associated with a better retention of secukinumab at 1 year.

**Conclusion:**

Following its approval in France, ~59% of axSpA patients retained secukinumab in daily practice, at 1 year. Prior exposure to TNFi, OSI and IBD were identified as risk factors for secukinumab discontinuation.

**What is already known on this topic?**

- Secukinumab, a fully human monoclonal antibody to interleukin-17A, is approved for the treatment of axial spondyloarthritis (axSpA); however, there is a lack of real-world data.

**What this study adds?**

- In real-world setting, treatment with secukinumab showed 59% retention rate after 1 year of treatment in the French patients with axSpA.
- Prior exposure to biologic disease-modifying agents, the presence of objective signs of inflammation, and past or present history of inflammatory bowel disease were identified as high-risk predictive factors of secukinumab discontinuation.

**How this study might affect research, practice or policy?**

- This study will help in understanding the impact of patient characteristics, diagnosis or drug use (line of treatment or dosage) on secukinumab persistence in patients with axSpA under real-world settings.
- Also, the study will help researchers learn more about the predictive factors that influence secukinumab discontinuation in axSpA patients.

## Introduction

Axial spondyloarthritis (axSpA) is an inflammatory rheumatic disease that affects the axial skeleton (spine and/or sacroiliac joints), leading to severe pain, stiffness and fatigue.^1^ Various types of lymphoid and non-lymphoid cells (producing proinflammatory cytokines such as tumour necrosis factor (TNF)-α, interleukin (IL)−17, IL-23, etc) have been shown to play a combined role in the pathogenesis of axSpA.^2^ The proinflammatory cytokine, IL-17A, has been identified as a relevant therapeutic target for some chronic inflammatory disorders, including axSpA.^3 4^

Secukinumab is the first-in-class human monoclonal IgG1κ antibody that directly inhibits IL-17A and is approved for the treatment of patients with axSpA (radiographic (r-axSpA) and non-radiographic (nr-axSpA)).^5^ Secukinumab has demonstrated significant long-term efficacy and safety versus placebo in patients with axSpA across various randomised clinical trials (RCTs).^6–10^

RCTs evaluate sophisticated outcome measures including MRI, health-related quality of life with a stringent prospective, randomised and controlled design.^11 12^ However, RCTs typically have a smaller and well-defined study population. Data from RCTs may not fully mimic secukinumab treatment in a real-world setting because clinical trials are highly regulated and do not inevitably represent everyday practice. Moreover, close monitoring of patients with a predefined time for different visits at the centres is far from the daily practice. Therefore, conventional RCT outcomes may be inappropriate in the specific scenario of real-world data collection. Despite some limitations associated with real-world evidence (RWE) studies, such as analysis design and incomplete or missing data, these observational studies (prospective or retrospective) complement the evidence generated by RCTs and depend on everyday therapeutic use in the real-world setting.^13–15^ RWE studies can inform the application of RCTs to healthcare decision-making and provide insights beyond what RCT covers.^16^

Under real-world conditions, drug retention provides critical information on efficacy, safety, compliance and convenience of use. The retention of secukinumab has been evaluated in retrospective observational series and registries^17–22^ including axSpA.^20 23 24^ Although the factors influencing anti-TNF efficacy and retention in axSpA—such as smoking status, young age, gender, human leucocyte antigen (HLA)-B27 positivity, radiographic status, objective signs of inflammation (OSI) and rank of drug administration—have been identified in various RCTs and RWE studies,^25–27^ data on anti-IL17 agents are sparse.^20 22 23^ The impact that patient characteristics, diagnosis or drug use (line of treatment or dosage) might have on secukinumab retention under real-life conditions in patients with axSpA is only partially understood. This non-interventional retrospective study was designed to evaluate if the presence of OSI at the initiation of secukinumab is predictive of secukinumab retention at 1 year. The study also evaluated other predictive factors of retention in patients treated for active axSpA in France.

## Methods

### Study design and patients

FORSYA is an ongoing, multicentric, non-interventional, retrospective and descriptive study in adult axSpA and psoriatic arthritis (PsA) patients who were initiated on commercial secukinumab treatment between its launch (11 August 2016) and 31 August 2018. In this manuscript, we report the results obtained in the axSpA subgroup. Patients who received secukinumab for indications other than axSpA or PsA, patients who received secukinumab as an investigational medical product during an interventional trial, patients for whom no follow-up by the centre was available after secukinumab initiation, and patients who objected to the collection and use of data for this study were excluded.

The primary objective of this study was to evaluate if the presence of OSI at secukinumab initiation was associated with secukinumab retention at 1 year. In order to avoid a bias due to the non-evaluation of patients who had to stop their treatment very soon after its initiation, only the centres that were able to provide an exhaustive list issued from their electronic health record system or from their own specific databases of patients fulfilling the inclusion/exclusion criteria were involved in the study. The data were retrospectively collected from patients’ files (patient chart review study) between October 2019 and September 2020 by either a physician or research nurse at each centre or by an independent clinical research assistant from the Contract Research Organization.

The study was registered with Health Data Hub^28^ and conducted in accordance with the Guidelines for Good Pharmacoepidemiology Practices of the International Society for Pharmacoepidemiology (2015).^29^ All patients were individually informed of this study and had the opportunity to refuse the extraction of the data contained in their medical files.

### Assessments

The presence of OSI was predefined for axSpA patients by at least one of the criteria: a C reactive protein (CRP) level ≥5 mg/L within the 3 months before initiation of secukinumab or erythrocyte sedimentation rate (ESR) ≥28 mm/hour at secukinumab initiation or MRI inflammation at the sacroiliac or spine level (as defined by the local radiologist or rheumatologist) at any time before secukinumab initiation. Moreover, for the patients with a positive MRI, we have collected the date of the last MRI prior to secukinumab initiation. Presence of structural damage at the sacroiliac joints (SIJ) level on pelvic X-Rays was recorded in two different ways (a) by asking the physician whether she/he was initiating the drug because of an nr-axSpA or a r-axSpA (b) the fulfilment or not of the modified New York (mNY) criteria. Presence of MRI abnormality was only considered for the inflammation domain and was also recorded in two different ways (a) the fulfilment or not of the Assessment of SpondyloArthritis International Society (ASAS) 2009 criteria (b) the presence of inflammation based on the local investigator/radiologist opinion at MRI SIJ or Spine level before initiating the drug. The retention period of treatment was analysed as a function of time. This period was defined as the time interval between the start of secukinumab treatment and the final discontinuation of treatment. The candidate baseline predictive factors for secukinumab retention included sociodemographic data (age, gender, body mass index (BMI) and tobacco status), axSpA anamnesis (disease duration, radiographic structural damage on pelvic X-rays or inflammation on MRI, HLA-B27, history of synovitis, enthesitis, uveitis, inflammatory bowel disease (IBD) and psoriasis) and comorbidities (eg, cardiovascular disease, hypertension, metabolic syndrome, renal insufficiency, severe infections, gastroduodenal ulcers, osteoporosis, depression, fibromyalgia, cancer) and OSI. The treatment regimen of secukinumab, the loading and maintenance doses at initiation, treatment modifications and the reasons for treatment modifications and concomitant treatment at initiation were also collected and assessed.

### Statistical analysis

The mean and SD were used to describe quantitative variables and were reported in terms of absolute frequency and percentage by modality for 95% CI of the percentages. Cox proportional hazard regression models (univariate and multivariate) were applied to investigate the predictive factors at secukinumab initiation affecting the retention at 1 year of secukinumab treatment. The dependent variable was the time to secukinumab definitive discontinuation within 1 year, meaning that in case of temporarily discontinuation with a reinitiation of the drug later on the status of the patient at the end of the second period was considered. Any data that were ‘not available’ were considered as ‘missing’; hence, these data were not considered when computing the proportion of patients per modality in the qualitative variable analysis. For predictive factors with less than 20% of missing data, a multiple imputation approach was applied using the method developed by Rubin^30^ and data were entered in a multivariate model using a stepwise selection (significance level for entering variables=20%; significance level for removing variables=10%). OSI was forced into the model regardless of its significance level or rate of missing data. The time to definitive secukinumab discontinuation and retention were analysed using a Kaplan-Meier (KM) analysis. Survival probability estimates at 6 months and 1 year were calculated with 95% CIs using the log–log transformation. Moreover, as a post hoc analysis, we have evaluated the percentage of patients still on treatment at 1 year with regards to the time of the MRI assessment (within 3, 6, 12 months or more than 12 months prior the baseline visit). The percentage values of discontinuation due to intolerance were calculated against total discontinued patients (n=476). The analyses were performed using SAS software V.9.4 or higher.

## Results

### Patient and study course

**Figure 1:**
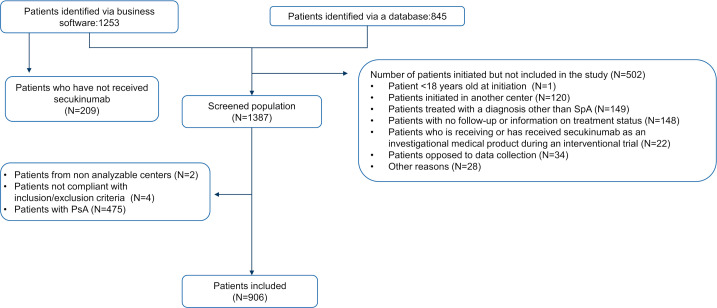
Patient and study course. N, size of the population; PsA, psoriatic arthritis; SpA, spondyloarthritis.

Among the 48 active centres that included patients in this study, 47 fulfilled the criteria related to exhaustivity of information about their centre’s patients receiving secukinumab. A total of 2098 patients were identified, 59.7% through electronic health records and 40.3% through each centre’s personal database. Among the patients identified, 34.2% did not meet eligibility criteria and were excluded (primarily because of other diagnosis or the absence of follow-up) and 1381 patients were eligible. This study reports the results observed in the groups of patients where secukinumab was initiated based on a diagnosis of axSpA (685 with r-axSpA and 221 with nr-axSpA) (figure 1).

### Baseline demographics and disease characteristics

Demographics and disease characteristics of the analysed patients are summarised in table 1. Overall, the mean (SD) age was 46.2 (11.7) years, the mean BMI was 27.0 kg/m^2^, and approximately 42% of the patients were men. At secukinumab initiation, 67.7% of patients were non-smokers. The mean (SD) disease duration was 9.3 (9.1) years; the shortest and longest durations were observed in the first line (4.6 years) and in the ≥third line (10.2 years) treatment groups, respectively. More than 60% of patients were HLA-B27 positive, 25% had concomitant psoriasis and 78% had evidence of radiologic (MRI or X-rays) signs of axSpA on the sacroiliac joint. Based on the opinion of the investigator, 685 and 221 patients were suffering from a radiographic and a non-radiographic axSpA, respectively. Among the 685 r-axSpA patients, an information related to the mNY criteria was available in 405 and a fulfilment of the mNY criteria for presence of SIJ structural damage was noticed in 323 (80%). The presence of inflammation at MRI was observed in (a) 401 out of 491 (82%) patients with information on the fulfilment of the ASAS criteria for the presence of inflammation at SIJ-MRI b) 488 out of 703 (69%) patients when considering the presence of inflammation at MRI of either the SIJ or the spine before the initiation of secukinumab. At least one OSI was reported in 86.3% of patients (41.3% had a CRP level ≥5 mg/L (or an ESR ≥28 mm/hour) and 69.4% had an MRI sign of inflammation on the sacroiliac joint or the spine). It has to be mentioned that objective sign of inflammation was absent not only in 14% of the patients with a radiographic status of their disease but also in 24% of the patients with a non-radiographic status of their disease, condition which is in contradiction with the current recommendation of use of biotherapy in nr-axSpA. Secukinumab was the first line of treatment in 72 patients (8%), second line in 134 patients (14.9%) and third or subsequent lines in the majority of patients, that is, in 693 patients (77.1%) (data not available for 7 patients). In the third-line treatment group, the mean (SD) number of biologic/targeted synthetic disease-modifying antirheumatic drugs (b/tsDMARDs) previously received was 3.3 (1.4). Other than anti-TNF, 40 patients had been treated with ustekinumab; 14 with tocilizumab; 11 with abatacept and<5 with rituximab, ixekizumab and apremilast.

**Table 1:** Baseline demographics and disease characteristics

Parameters	Total N=906	OSI+ N=617	OSI- N=98	nr-axSpA N=221	r-axSpA N=685	First line* N=72	Second line* N=134	≥Third line* N=693
Age (years), mean (SD); m	46.2 (11.7); m=906	45.6 (11.6); m=617	47.6 (12.5); m=98	45.9 (11.2); m=221	46.3 (11.9); m=685	41.7 (12.6); m=72	46.5 (12.2); m=134	46.6 (11.4); m=693
Male, n/m (%)	382/906 (42.2)	267/617 (43.3)	37/98 (37.8)	58/221 (26.2)	324/685 (47.3)	30/72 (41.7)	69/134 (51.5)	279/693 (40.3)
BMI (kg/m^2^), mean (SD); m	27.0 (5.5); m=533	27.2 (5.7); m=387	25.9 (5.2); m=52	26.5 (5.2); m=128	27.1 (5.6); m=405	25.5 (4.4); m=45	26.5 (5.2); m=81	27.2 (5.7); m=405
Current smoker, n/m(%)	231/715 (32.3)	169/501 (33.7)	21/71 (29.6)	55/173 (31.8)	176/542 (32.5)	29/61 (47.5)	28/110 (25.5)	174/539 (32.3)
Disease duration, years, mean (SD); m	9.3 (9.1); m=807	8.4 (8.7); m=556	11.3 (9.7); m=85	6.5 (6.9); m=197	10.3 (9.6); m=610	4.6 (7.9); m=66	7.6 (8.7); m=120	10.2 (9.1); m=615
HLA-B27 +, n/m (%)	527/825 (63.9)	363/571 (63.6)	49/91 (53.8)	98/197 (49.7)	429/628 (68.3)	38/66 (57.6)	77/124 (62.1)	406/629 (64.5)
Sacroiliac joint radiographic status								
nr-axSpA, n/m (%)	221/906 (24.4)	139/617 (22.5)	43/98 (43.9)	221/221 (100.0)	NA	15/72 (20.8)	33/134 (24.6)	171/693 (24.7)
r-axSpA, n/m (%)	685/906 (75.6)	478/617 (77.5)	55/98 (56.1)	NA	685/685 (100.0)	57/72 (79.2)	101/134 (75.4)	522/693 (75.3)
MRI sacroiliitis according to the ASAS definition; n/m (%)	401/491 (81.7)	397/441 (90.0)	0/14 (0.0)	83/95 (87.4)	318/396 (80.3)	50/53 (94.3)	61/74 (82.4)	289/363 (79.6)
Past or present arthritis/synovitis, n/m (%)	258/803 (32.1)	163/544 (30.0)	33/91 (36.3)	64/196 (32.7)	194/607 (32.0)	13/69 (18.8)	38/119 (31.9)	206/611 (33.7)
Past or present enthesitis, n/m (%)	314/756 (41.5)	221/520 (42.5)	29/92 (31.5)	67/178 (37.6)	247/578 (42.7)	14/64 (21.9)	46/110 (41.8)	254/579 (43.9)
Past or present extra-rheumatological manifestations								
IBD, n/m (%)	22/878 (2.5)	17/599 (2.8)	1/96 (1.0)	3/212 (1.4)	19/666 (2.9)	0/70 (0.0)	1/130 (0.8)	21/672 (3.1)
Psoriasis, n/m (%)	199/791 (25.2)	140/549 (25.5)	20/88 (22.7)	56/196 (28.6)	143/595 (24.0)	8/63 (12.7)	27/124 (21.8)	163/599 (27.2)
Uveitis, n/m (%)	131/859 (15.3)	86/583 (14.8)	9/94 (9.6)	22/207 (10.6)	109/652 (16.7)	7/69 (10.1)	22/129 (17.1)	100/655 (15.3)
At least one OSI, n/m (%)	617/715 (86.3)	–	–	139/182 (76.4)	478/533 (89.7)	63/68 (92.6)	86/100 (86.0)	463/542 (85.4)
CRP≥5 mg/L or ESR≥28 mm/h at secukinumab initiation, n/m (%)	282/683 (41.3)	–	–	67/171 (39.2)	215/512 (42.0)	30/64 (46.9)	35/99 (35.4)	214/514 (41.6)
MRI OSI at the sacroiliac joint or spine, n/m (%)	488/703 (69.4)	–	–	108/186 (58.1)	380/517 (73.5)	58/65 (89.2)	73/104 (70.2)	353/530 (66.6)

*Information for some axSpA patients was missing in this retrospective analysis.

ASAS, Assessment of SpondyloArthritis International Society; BMI, body mass index; CRP, C reactive protein; ESR, erythrocyte sedimentation rate; HLA-B27, human leucocyte antigen B-27; IBD, inflammatory bowel disease; m, number of samples; N, size of the population; n/m, number with event/number of patients; nr-axSpA, non-radiographic axial spondyloarthritis; OSI, objective signs of inflammation; r-axSpA, radiographic axial spondyloarthritis.

### Secukinumab treatment

At initiation, 95% of patients received loading dose of secukinumab, of which 86.4% received 150 mg dose and 9.7% received 300 mg dose every week for the first 4 weeks. After 4 weeks, 82.8% and 9.1% of the patients received maintenance dose of secukinumab 150 mg and 300 mg every 4 weeks, respectively. After secukinumab initiation, an increase in the dosage was required in 164 patients (18.1%) due to insufficient efficacy and in 60 patients (6.6%) due to end-dose effect. At the time of secukinumab initiation, 48.6% of patients were on non-steroidal anti-inflammatory drugs (NSAIDs) (since the onset of the disease 92.2% (819/888) of the patients received at least once a NSAID for their axSpA (mean number of NSAIDs used : 3.3±18)), 11.6% on conventional synthetic disease-modifying antirheumatic drugs and 7.3% on corticosteroids as concomitant treatment.

### Secukinumab retention rate

**Figure 2:**
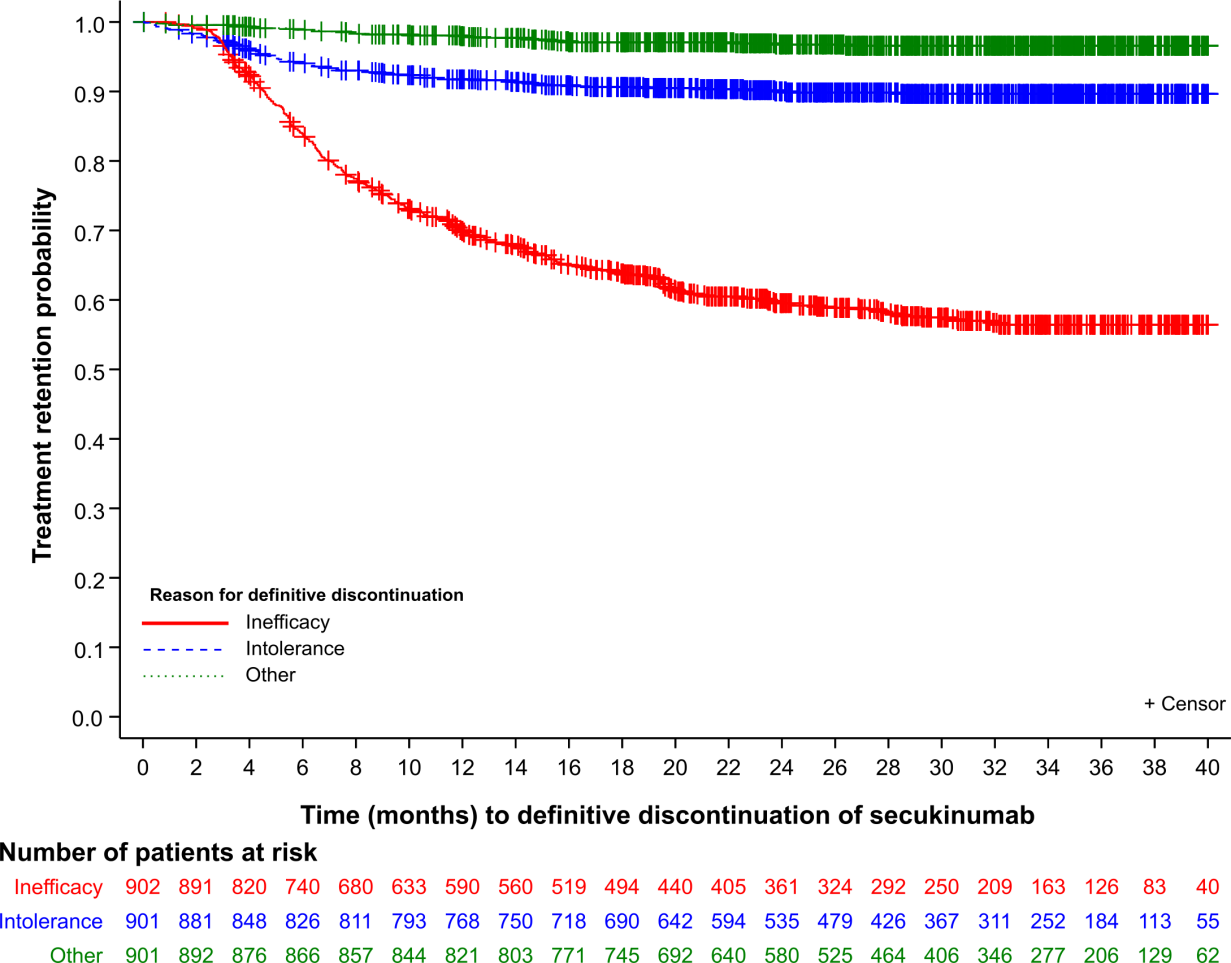
Secukinumab retention rate with event (discontinuation) defined by inefficacy or intolerance or other reason.

After starting secukinumab treatment, the mean (SD) follow-up duration was 844.7 (294.4) days. The mean (SD) time to definitive discontinuation of secukinumab was 274.7 (200.9) days and the number of patients who discontinued treatment was 476. The majority of treatment discontinuations were due to inefficacy (n=360; 75.6%), intolerance (n=88; 18.5%) and other reasons (n=28; 5.9%) (figure 2). The main reasons for discontinuation due to intolerance included infection (n=22; 4.6%), allergy (n=8; 1.68%), IBD (n=3; 0.63%) and uveitis (n=2; 0.42%). The percentage values of discontinuation were calculated against the total number of patients that discontinued treatment (n=476). The KM retention rate for secukinumab was 76% (95% CI 74 to 79%) at month 6 and 59% (95% CI 55 to 62%) at month 12, with no differences observed between r-axSpA and nr-axSpA patients.

### Predisposing factors of secukinumab retention rate

#### Univariate Cox regression

The hazard of definitive discontinuation of secukinumab at 1 year was 1.20 times higher (95% CI 0.84 to 1.72) in patients with at least one OSI versus patients without OSI. The difference was not statistically significant (p=0.316) (table 2). Past or present history of IBD (Crohn’s disease or haemorrhagic rectocolitis) (HR: 1.96 (95% CI 1.13 to 3.41); p=0.017) and ≥third line of secukinumab treatment (HR: 1.69 (95% CI 1.08 to 2.66); p=0.023) were the only significant factors associated with secukinumab treatment discontinuation at 1 year (table 2).

**Table 2:** Predisposing factors of secukinumab discontinuation at 1 year (univariate analysis)

N=903†		% patients still on treatment at 1 year	Univariate Cox regressions		
Predictive factor	Modality* (N)		HR (95% CI)	P value‡	P type III
At least one objective sign of inflammation	No = (N=97)*	65.3%	1.20 (0.84 to 1.72)	0.316	
	Yes (N=616)	58.8%			
Age (years)	≤40 (N=288)*				0.231
	>60 (N=108)	59.3%	0.90 (0.64 to 1.27)	0.550	
	40–60 (N=507)	61.3%	0.82 (0.66 to 1.03)	0.088	
Gender	Male (N=380)*	50.9%			
	Female (N=523)	59.2%	1.03 (0.84 to 1.27)	0.776	
BMI (kg/m^2^)	Normal weight (≥18.5 and <25) (N=200)*	64.2%			
	Obesity (≥30) (N=133)	63.4%	1.03 (0.72 to 1.48)	0.870	0.944
	Pre-obesity (≥25 and <30) (N=186)	60.8%	1.09 (0.79 to 1.51)	0.590	
	Underweight (<18.5) (N=12)	66.7%	0.90 (0.33 to 2.47)	0.840	
Smoking status (at secukinumab initiation)	Never (N324)*	61.0%			0.595
	Former smoker (N=158)	60.8%	1.02 (0.75 to 1.38)	0.912	
	Current smoker (N=231)	57.4%	1.14 (0.88 to 1.48)	0.328	
Diagnosis delay (years)	≤2.5 (N=289)*	59.3%			0.106
	2.5 to ≤5 (N=89)	71.1%	0.63 (0.41 to 0.97)	**0.034**	
	<5 (N=179)	59.8%	0.91 (0.68 to 1.22)	0.537	
Disease duration (years)	≤5 (N=345)*	57.7%			0.573
	5 to ≤(N=192)	61.9%	0.88 (0.67 to 1.17)	0.385	
	>10 (N=268)	60.4%	0.89 (0.70 to 1.15)	0.378	
HLA-B27 positivity	No (N=297)*	60.1%			
	Yes (N=525)	58.8%	1.05 (0.84 to 1.32)	0.652	
Radiological structural damage according to mNY criteria	No (N=155)*	55.8%			
	Yes (N=325)	58.9%	0.94 (0.70 to 1.25)	0.666	
CRP≥5 mg/L or ESR≥28 mm if CRP not available	No (N=398)	59.1%			0.42
	Yes (N=282)	62.4%	0.90 (0.71 to 1.16)	0.422	
Sign of inflammation in the MRI of the sacroiliitis or spine regardless of the date prior to the initial prescription of secukinumab	No (N=214)	66.0%			0.06
	Yes (N=487)	57.4%	1.29 (0.99 to 1.68)	0.063	
Past or present history of active arthritis/synovitis diagnosed by a doctor	No (N=543)	59.3%			
	Yes (N=258)	62.8%	0.88 (0.69 to 1.11)	0.278	
Past or present history of psoriasis	No (N=589)*	60.6%			0.660
	Yes (N=199)	58.3%	1.06 (0.82 to 1.36)	0.063	
Past or present history of uveitis	No (N=725)*	59.6%			
	Yes (N=131)	61.8%	0.92 (0.68 to 1.24)	0.576	
Past or present history of IBD (Crohn’s disease or haemorrhagic rectocolitis)	No (N=853)*	59.8%			
	Yes (N=22)	40.9%	1.96 (1.13 to 3.41)	**0.017**	
Secukinumab maintenance dose at initiation (per month)	150 mg (N=747)*	61.1%			0.940
	300 mg (N=88)	62.5%	0.94 (0.65 to 1.34)	0.727	
	Other (N=5)	100%	<0.01 [0.01 to >999.99)	0.963	
Secukinumab treatment line	First line (N71)*	72.2%			0.062
	Second line (N=133)	62.7%	1.49 (0.89 to 2.51)	0.129	
	≥Third line (N=692)	57.6%	1.69 (1.08 to 2.66)	**0.023**	
Concomitant treatment with csDMARDs at initiation	No (N=758)*	59.4%			
	Yes (N=145)	60%	0.93 (0.70 to 1.23)	0.604	
Oral corticosteroids intake at initiation of secukinumab	No (N=724)*	60.5%			
	Yes (N=57)	63.2%	0.90 (0.58 to 1.40)	0.633	
History of depression or anti-depressive concomitant treatment	No (N=703)*	60.8%			
	Yes (N=165)	54.5%	1.25 (0.97 to 1.61)	0.089	
History or suspicion of fibromyalgia	No (N=787)*	60.4%			
	Yes (N=89)	52.8%	1.25 (0.90 to 1.72)	0.181	
History of depression or anti-depressive concomitant treatment or history or suspicion of fibromyalgia	No (N=641)*	61.0%			
	Yes (N=226)	55.3%	1.22 (0.97 to 1.54)	0.090	
Concomitant treatment with a PPI	No (N=609)*	59.4%			
	Yes (N=246)	59.5%	0.99 (0.78 to 1.25)	0.931	

*The modality given in the first row (eg, ‘No’ for ‘At least one objective sign of inflammation’) defined the reference in the Cox model.

†Although the study included 906 axSpA patients, the Cox univariate analysis included 903 patients, since the time (days) to definitive discontinuation of secukinumab within the first year (≤365 days) could not be calculated for three patients.

‡ P value calculated against reference value.

axSpA, axial spondyloarthritis; BMI, body mass index; csDMARDs, conventional synthetic disease-modifying anti-rheumatic drugs; HLA-B27, human leucocyte antigen B-27; IBD, inflammatory bowel disease; mNY, modified New York; N, size of the population; PPI, proton-pump inhibitor.

A post hoc analysis evaluating specifically the impact of the line of therapy on secukinumab retention rate with regards to the presence (vs absence) of OSI showed the following (values given are number and (%) of patients still on treatment at 1 year in the group of patients with versus without OSI, respectively).

1. no clear association when the drug was prescribed as first-line biologic therapy (45 (71%) vs 4 (80%), HR=1.81 (95% CI 0.24 to 13.54); p value=0.565).
2. a trend to a better retention rate when the drug was initiated as second-line biologic therapy (60 (70%) vs 8 (57%), HR=0.63 (95% CI 0.26 to 1.54); p=0.312).
3. a trend to a worse retention rate when the drug was initiated as a third or more biologic therapy (255 (55%) vs 52 (66%), HR=1.35 (95% CI 0.90 to 2.02); p=0.141).

#### Multivariate Cox regression

After multiple imputation, at least one OSI (HR: 1.44 (95% CI 1.08 to 1.93); p=0.014), past or present history of IBD (HR: 1.76 (95% CI 1.01 to 3.07); p=0.047) and ≥third line of secukinumab treatment (HR: 1.67 (95% CI 1.06 to 2.62); p=0.028) and depression (HR:1.25 (0.97 to 1.60); p=non-significant) were predictors of secukinumab treatment discontinuation at 1 year (table 3).

**Table 3:** Predisposing factors of secukinumab discontinuation at 1 year (multivariate analysis)

Predictive factor	Modality* (N)	Multivariate Cox regression		
		HR (95% CI)	P value†	P type III
At least one objective sign of inflammation	No (N=165)*			
	Yes (N=711)	1.44 (1.08 to 1.93)	**0.014**	
Secukinumab treatment line	First line (N=68)*			0.084
	Second line (N=132)	1.53 (0.91 to 2.57)	0.107	
	≥Third line (N=676)	1.67 (1.06 to 2.62)	**0.028**	
Past or present history of IBD	No (N=854)*			
	Yes (N=22)	1.76 (1.01 to 3.07)	**0.047**	
History of depression or anti-depressive concomitant treatment	No (N=716)*			
	Yes (N=160)	1.25 (0.97 to 1.60)	0.090	

*The modality given in the first row (eg, ‘No’ for ‘At least one objective sign of inflammation’) defined the reference in the Cox model.

†P value calculated against the reference value. Factors with >20% missing data such as age, gender, BMI, smoking status, duration of disease, axial involvement, past or present history of synovitis, psoriasis, uveitis, and fibromyalgia, initial secukinumab dose, concomitant treatment with csDMARDs, oral corticosteroids, HLA-B27 positivity and sacroiliitis were not included in the multivariate Cox analysis.

BMI, body mass index; csDMARDs, conventional synthetic disease-modifying anti-rheumatic drugs; HLA-B27, human leucocyte antigen B-27; IBD, inflammatory bowel disease; N, size of the population.

#### KM curve

**Figure 3:**
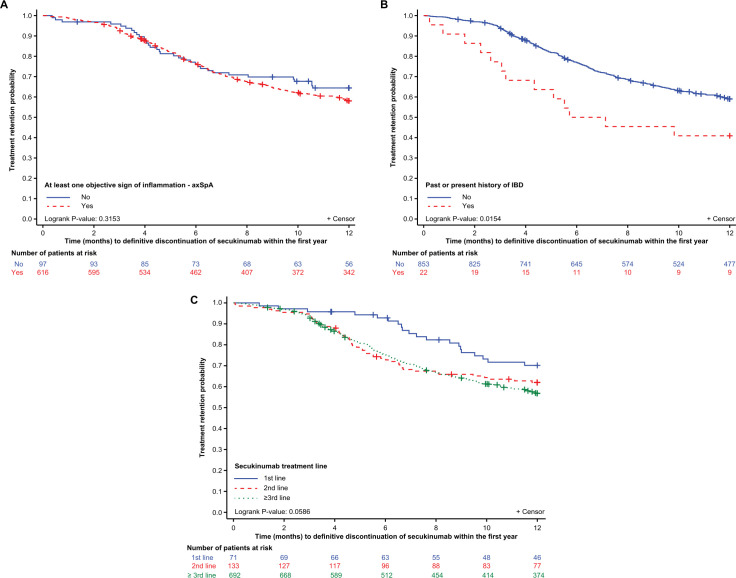
Kaplan-Meier analysis of time to definitive secukinumab discontinuation defined by (A) OSI. (B) Past or present History of IBD. (C) Line of treatment. axSpA, axial spondyloarthritis; IBD, inflammatory bowel disease; OSI, objective sign of inflammation.

The 1-year KM retention of secukinumab according to predictive factors identified in the Cox multivariate analysis is presented in figure 3. The 1-year KM retention rate for secukinumab was 58% (95% CI 54 to 62%) and 64% (95% CI 54 to 73%) (p=0.315) for patients with or without OSI, respectively; 41% (95% CI 21 to 60%) and 59% (95% CI 56 to 62%) (p=0.015) for patients with or without IBD. The 1-year KM retention rate for secukinumab was numerically greater in first line versus second and ≥third line (70% (95% CI 59% to 81%), 62% (95% CI 54% to 70%) and 57% (95% CI 53 to 61%); (p=0.059), respectively) treatment groups.

#### Retention rate with regards to the time of MRI assessment

In the subgroup of patients with presence of inflammation at MRI and for whom this information was available (n=402 patients), the pourcentage of patients still on treatment was 65%, 66%, 59% and 56% in case this MRI had been performed within 3, 6, 12 months and more than 12 months prior baseline visit.

### Safety

During the treatment period, 186 patients (20.5%) had at least one adverse event related to secukinumab, which led to treatment adaptation for 79 (8.7%) patients, hospitalisation for 22 (2.4%) patients and to treatment discontinuation for 121 (13.4%) patients. As expected, infections and infestations were the most common adverse events (10.4%), followed by gastrointestinal disorders (4.6%). Of these gastrointestinal disorders, there were three cases of Crohn’s disease, and all three of these required treatment discontinuation; hospitalisation was also required in one case and there was also one case of haemorrhagic rectocolitis. In addition, uveitis was reported in seven cases and led to therapy discontinuation in four of these cases.

## Discussion

This study evaluated the rate of retention of secukinumab in real-world practice at the time it was approved in France and made it possible to highlight certain factors associated with this drug retention. Treatment with secukinumab showed a 59% retention rate after 1 year of treatment in patients mostly refractory to biological therapy under real-world conditions. The main cause of discontinuation in our study was lack of efficacy. Prior exposure to b/tsDMARD, OSI and past or present history of IBD were identified as predictive factors of secukinumab discontinuation.

The previously reported 1-year retention rate of secukinumab in real-world settings was in the range of 61%–79% in both axSpA and PsA patients.^17 19 20 31^ In comparison to these findings, retention rate in the current study was slightly lower, presumably because large proportion of patients received secukinumab as ≥third line of therapy. Furthermore, based on literature, the retention rate of anti-TNF agents in the French population is lower than in other countries.^32^ This could be explained by the less restrictive access to treatment in France, facilitating faster treatment switches. A disparity of secukinumab retention rates between countries has also been observed in the European Spondyloarthritis Research collaboration Network study.^20 33^

In various RCTs and RWE studies of axSpA patients, the factors reported to influence anti-TNF therapy efficacy and retention rate are smoking status, young age, gender, HLA-B27 positivity, radiographic status, OSI and rank of drug administration.^24 25^ This study aimed to evaluate whether OSI is associated with secukinumab retention and to determine if other predictive factors could be identified. The main predictive factors associated with secukinumab discontinuation were the presence of OSI, line of treatment and history of IBD. Based on the univariate Cox regression analysis, at least one OSI at 1 year was not a significant predictor of secukinumab discontinuation; however, after multiple imputation, the multivariate analysis revealed that OSI was a significant predictor of secukinumab discontinuation. This result was unexpected as it was observed that patients with active inflammatory disease were more likely to benefit from anti-TNF.^25^ Although >20% of the data for OSI were missing, the variable was forced into the multivariate model irrespective of its significance level or rate of missing data. In this study, the majority (>85%) of patients presented with OSI at treatment initiation, which might also impact the analysis. Moreover, in the MEASURE trials that assessed efficacy of secukinumab in patients with r-axSpA, even if a good response was observed in patients with a CRP level <5 mg/L, the magnitude of response to secukinumab was higher in patients with an elevated CRP level ≥5 mg/L.^34^ Another reason to explain this unexpected result was the fact that in this analysis, we have defined a « positive » MRI by any MRI with presence of inflammation whenever this MRI had been performed. The post hoc analysis performed in the subgroup of patients with positive MRI and with the availability of the date of the MRI with regards to the initiation of secukinumab suggested a better retention rate at 1 year in case of a recent positive MRI (65% vs 56% in case an MRI performed within 6 months vs more than 12 months prior secukinumab initiation, respectively. However, one could also explain these results (absence of OSI as a predisposing factor of a better drug retention rate) by the fact that secukinumab at the time of its launch in France has been considered as the last opportunity for a lot of patients and had been continued in a longer run in patients without objective sign of inflammation than in the patients with presence of OSI. In this latter group a re-initiation of a previous biotherapy and/or another anti-TNF could have been proposed. Finally, the divergence between the results of the uni- versus multi-variate analyses and also the narrow difference between these two groups (see figure 3C) might also suggest that this statistically significant difference is without any clinical relevance. The additional analyses conducted in different sub-groups of patients with regard to the line of therapy and the presence vs absence of OSI are questionable because of 1) the low number of patients in some categories and 2) the high percentage of patients with OSI.

It is well established that the retention rate of secukinumab is the highest when this therapy is used as the first biological agent in patients with axSpA or PsA compared with when it is used as second or third line of treatment,^20 22 35^ likewise with any b/tsDMARD. In alignment with other real-world studies, better retention of secukinumab in first-line treatment compared with ≥third-line treatment (70% vs 57%) was observed in this study and ≥third line of secukinumab was a significant predictor of secukinumab discontinuation at 1 year.^17 35^

Past or present history of IBD was found to be a significant predictor of secukinumab discontinuation. Contrary to anti-TNF therapies, secukinumab is known to be ineffective in IBD. It is established that there is a low incidence rate of developing new-onset IBD or exacerbating preexisting IBD with anti-IL-17 therapy.^36^ In generally, patients with IBD who discontinue anti-TNF therapy tend to experience a clinical relapse.^36–39^ As this study included patients with history of IBD who were previously on anti-TNF therapy and then switched to secukinumab, it is possible that they experienced a relapse of IBD after discontinuation of anti-TNF therapy, which then led to discontinuation of secukinumab. A history of depression was also identified as a predictor of secukinumab discontinuation although the effect was not significant, consistent with the results reported in the Cantabria and ASTURias study.^17^

In contrast to anti-TNF therapies, gender, young age, HLA-B27 positivity and radiographic status were not predictors of secukinumab retention in this study. The findings on gender were consistent with the MEASURE studies, where efficacy and safety outcomes were comparable between male and female axSpA patients treated with secukinumab over 52 weeks.^40^ In the real-word German AQUILA study in r-axSpA, the secukinumab retention rate between male and female patients was not significantly different.^41^ In pooled analyses of the MEASURE studies, secukinumab was effective in patients with r-axSpA regardless of their HLA-B27 status; however, patients who were HLA-B27 positive seemed to derive increased therapeutic benefit than those who were HLA-B27 negative.^42^ Other than the aforementioned factors, baseline patient characteristics did not have a major impact on the overall secukinumab retention in this study.

Overall, secukinumab treatment was well tolerated in patients with axSpA. Throughout the treatment period, no new or unexpected safety signals were observed. The safety profile was consistent with the established safety profile across approved indications.^43^

The strength of this study lies in the fact that the findings complement clinical trial results. RWE studies provide valuable data on the predictive factors for retention, safety and survival of secukinumab in a heterogeneous French patient population with comorbidities, which is not commonly reported in the RCT. Furthermore, the study enrolled a larger patient population than previously published studies.^17 19 31^

This study has some limitations; due to the retrospective collection of patient data exclusively from that available in the source record, the number of data gaps inherent in this type of study was inevitable. The anticipated high frequency of missing data concerning several parameters/items was the main reason of the choice of drug retention rate as the primary outcome in this study since we anticipated that this information (date of initiation and date of discontinuation of the drug) will be available in the majority of patients in contrast to other parameters/items. Therefore, despite we can be quite confident concerning this primary analysis (eg, % patients still on treatment overtime), the other analyses and in particular the evaluation of the predisposing factors of this retention rate can be more questionable. A small proportion of patients received secukinumab as the first or second-line treatment, whereas a larger number of patients received it as ≥third-line treatment. Also, a higher proportion of patients with r-axSpA received secukinumab compared with those with nr-axSpA. Several factors indicated as predictors of anti-TNF therapy retention, such as BMI and smoking status, could not be included in the multivariate Cox regression analysis because >20% of the data were missing. The high percentage of missing data concerning obesity and smoking could be also considered as a weakness of the study since these two patients’ characteristics (and in particular obesity) have been previously reported as predisposing factors of IL-17 retention rate in axSpA with conflicting results: obesity associated with a longer^44^ or a shorter retention rate. It is obviously important to check whether there is a difference in the predisposing factors of drug retention rate based on their mechanism of action to potentially guide the choice of the drug (IL17 vs TNF inhibitors) to use as the first biotherapy in daily practice.

For example, the fact that obesity has been reported with a better retention rate of IL-17-inhibitors might be explained by the IL-17-pathway since obesity has been shown to promote Th17 differentiation and IL-17 production^45^

However, the conflicting results observed in the different reported clinical studies regarding the impact of obesity on IL-17 inhibitors retention rate preclude any specific recommendation for the use of IL-17 inhibitors in this group of patients.

In summary, the overall retention of secukinumab in daily practice in the period following its approval in France was approximately 59% at 1 year in axSpA patients. Prior exposure to b/tsDMARDS, OSI and IBD was identified as predictive factors of secukinumab discontinuation. It might be of interest to replicate this study by evaluating this retention rate remotely from the launch of the molecule.

## Data Availability

Data are available upon reasonable request.
